# Coronavirus covid-19 detection by means of explainable deep learning

**DOI:** 10.1038/s41598-023-27697-y

**Published:** 2023-01-10

**Authors:** Francesco Mercaldo, Maria Paola Belfiore, Alfonso Reginelli, Luca Brunese, Antonella Santone

**Affiliations:** 1 Department of Medicine and Health Sciences “Vincenzo Tiberio”, University of Molise, Campobasso, Italy; 2 Department of Precision Medicine, University of Campania “Luigi Vanvitelli”, Naples, Italy

**Keywords:** Biological techniques, Radiography

## Abstract

The coronavirus is caused by the infection of the SARS-CoV-2 virus: it represents a complex and new condition, considering that until the end of December 2019 this virus was totally unknown to the international scientific community. The clinical management of patients with the coronavirus disease has undergone an evolution over the months, thanks to the increasing knowledge of the virus, symptoms and efficacy of the various therapies. Currently, however, there is no specific therapy for *SARS-CoV-2 virus, know also as Coronavirus disease 19,* and treatment is based on the symptoms of the patient taking into account the overall clinical picture. Furthermore, the test to identify whether a patient is affected by the virus is generally performed on sputum and the result is generally available within a few hours or days. Researches previously found that the biomedical imaging analysis is able to show signs of pneumonia. For this reason in this paper, with the aim of providing a fully automatic and faster diagnosis, we design and implement a method adopting deep learning for the novel coronavirus disease detection, starting from computed tomography medical images. The proposed approach is aimed to detect whether a computed tomography medical images is related to an healthy patient, to a patient with a pulmonary disease or to a patient affected with Coronavirus disease 19. In case the patient is marked by the proposed method as affected by the Coronavirus disease 19, the areas symptomatic of the Coronavirus disease 19 infection are automatically highlighted in the computed tomography medical images. We perform an experimental analysis to empirically demonstrate the effectiveness of the proposed approach, by considering medical images belonging from different institutions, with an average time for Coronavirus disease 19 detection of approximately 8.9 s and an accuracy equal to 0.95.

## Introduction

The SARS-CoV-2 virus enters the body by binding to the angiotensin 2 converting enzyme, an enzyme involved in blood pressure regulation and found on the cells of the lung epithelium where it defends the lungs from damage from infections and inflammation. The virus, by binding to ACE2, enters the cell and prevents the enzyme from fulfilling its protective role^1^.

Once in the cells, SARS-CoV-2 begins to replicate and clinically this phase is typically characterized by malaise, fever and dry cough^2^.

In some cases the SARS-CoV-2, also know as *covid-19* (acronym of the English COronaVIrus Disease 19) can evolve into a second phase which is characterized by alterations in the lungs with interstitial pneumonia^3^, very often bilateral and therefore with the involvement of both lungs, associated with respiratory symptoms that may initially be limited but which may lead to progressive clinical instability with respiratory failure^4^.

In a restricted number of patients, the clinical picture may worsen. As a matter of fact the infection leads to a state of excessive inflammation^5^, with local and systemic consequences (i.e., of the whole organism), with the risk of serious and sometimes permanent lung lesions (pulmonary fibrosis)^6^.

A clinical picture that can worsen further and lead to severe acute respiratory distress syndrome and sometimes to disseminated intravascular coagulation phenomena, with the formation of thrombus in small vessels throughout the body and the potential interruption of the normal flow of blood^7,8^.

In light of these three phases of the disease and also taking into account the radiological criteria, the US National Institutes of Health (NIH) have formulated a classification of the five clinical stages of *covid-19*^9,10^:

- *Asymptomatic or pre-symptomatic infection*: there is a diagnosis of SARS-CoV-2, but a complete absence of symptoms^11^ (but the subject is still contagious even in the absence of symptoms);
- *Mild illness*: the patient has mild symptoms (fever, cough, altered taste, malaise, headache, myalgia or muscle aches), but there is neither dyspnoea (breathing difficulties) nor radiologically detectable changes^12^;
- Moderate disease: the patient has a saturation—that is the oxygenation of the blood that is detected with an oximeter, greater than or equal to 94% and there is clinical or radiological evidence of pneumonia^13^;
- *Severe disease*: where one of the parameters is the saturation is less than 94%^14^;
- *Critical illness*: with respiratory failure, septic shock and/ or failure of one or more organs.

People can be infected with this disease through coughing and in general with direct contacts^4^.

To limit transmission, precautions must be taken^15^, such as maintaining a safety distance of at least 1.5 m, and maintaining correct hygiene behavior (periodically washing and disinfecting hands^16^, sneezing or coughing into a handkerchief or with the elbow bent and where necessary wear masks and gloves)^17^.

Medical imaging (for example, chest computed tomography) is able to show pneumonia in patients with *covid-19*^18^. For this reason, the World Health Organization published several additional diagnostic protocols (https://www.who.int/emergencies/diseases/novel-coronavirus-2019/technical-guidance/laboratory-guidance) for increase *covid-19* detection from medical images.

Currently, diagnostic tests for the confirmation of *covid-19* infection are performed in various public and private laboratories.

The tests currently available to detect SARS-CoV-2 infection are as follows:

- molecular test, which highlights the presence of genetic material of the virus. It is performed on a rhino-pharyngeal swab;
- antigen test, which highlights the presence of components of the virus. It is performed on a rhino-pharyngeal swab;
- traditional or rapid serological test, which shows the presence of antibodies against the virus. Serological tests are performed on venous sampling and capillary blood.

The main problem of these tests is represented by the fact that their results are typically available in some hour or also in days^19,20^.

As discussed in current literature in this context^21,22^ there is the possibility to better diagnose *covid-19* by exploiting radiological imaging: this is the reason in this paper we consider the possibility to diagnose the *covid-19* disease using medical images in particular, computed tomography (CT) have been used.

In last years research community demonstrate, with several studies^2,23,24^, that it is possible to detect pulmonary diseases by consider the analysis medical images with artificial intelligence techniques. In a nutshell artificial intelligence currently represents a research field aimed to build models starting from data. In last is adopted from both the industrial that academic researchers for the development of methods for assist experts in the medical images interpretation.

We exploit in the proposed paper the transfer learning i.e., a method aimed to adapt a model to a task different from the one for which it was initially trained. The intuition behind the transfer learning idea is the the knowledge learned in a certain context can be immediately reapplied to another context by “retuning”: in this way it is possible to avoid to retrain the model from scratch.

Transfer learning allows two results, the first one is represented by the reuse of the behavior of a network already trained to effectively extract features from input data and the second one is represented by limiting the processing to a significantly smaller number of parameters (corresponding to the last layers)^25,26^.

To support radiologists and pathologists in the task of screening the populations, we consider the usage of deep learning with the aim to understand whether there is the presence of *covid-19* in CT images by considering transfer learning. Moreover, one of the distinctive points of the proposed contribution is the automatically localise the infected lung areas. To do this, the network activation layers are exploited: in a nutshell we consider the CT lung areas considered y the trained model for prediction generation, with the aim to furnish a kind of prediction explainability. The idea behind the activation layers adoption is that they can be considered as an insight for the radiologist to visualise the areas of the CT exams which deserve an in-depth analysis.

Artificial intelligence for the *covid-19* identification was already explored in^4^ for covid-19 diagnosis. With respect to the work presented in^4^ is the adoption of CT images, which offer diagnostic potential far superior to x-rays^27–29^.

As a matter of fact, the difference between CT and X-rays is really significant. As a matter of fact, pathologists and radiologists typically analyse X-rays to detect bone dislocations and fractures, but also to detect tumors and pneumonia. However, CT scans are a kind of advanced X-ray devices that pathologists and radiologists use to better diagnose injury to internal organs.

X-ray machines can fail to diagnose issues with damage to muscle, and in general to soft tissue. Differently, with CT scans is possible to have evidence of these problems^28^. Furthermore, X-rays show a 2D representation of the tissue under analysis, while from the other side the CT shows a 3D representation. In CT exams a thin layer of the body is crossed by a highly collimated X-ray beam, produced by a tube that rotates around the patient, in a consensual manner to detectors placed beyond the patient. CT exams offer a layered visualization of the anatomical structures eliminates the problem of overlapping present in the x-ray examination (that can be reflected by noise in the data), revealing the presence of injury or disease to pathologists and radiologists. Furthermore, the approach proposed in^4^ considers two different step to detect the *covid-19* detection, while the proposed approach in a single step is able to detect whether a patient is affected by *covid-19*, a generic pulmonary disease or he/she is an healthy patient. Finally, while the method in^4^ is evaluated by considering a dataset freely available for research purposes, in this paper we evaluated the proposed approach with a dataset personally gathered and labelled by authors.

The idea behind the proposed method is the proposal of a deep learning network aimed to classify a pulmonary CT exams as related to a patient affected by the covid-19 disease, by other pulmonary disease or as healtly patient. The convolutional neural network is designed by authors. Moreover the proposed method is aimed to automatically localise the region of interest i.e., the areas of the image under analysis symptomatic of the covid-19 infection (for this task we resort to the grad-cam algorithm). Through the visualization of the areas of interest we are able to understand the reasons why the proposed model obtains a satisfactory accuracy. In fact, the proposed model is able to accurately localize the signs of the covid-19 in the medical image and is able to distinguish them from those of other lung diseases which, although giving similar symptoms, are not covid-19. The proposed model is able to understand this difference thanks to the use of different convolutional layers that allow it to extract different characteristics of the image at different levels of depth. In fact, tests made by the authors with convolutional models with a lower number of layers obtain unsatisfactory results. Convolutional layer consists of a collection of digital filters to perform the convolution operation on the input data, therefore by inserting more layers more features are extracted from the images and therefore it is possible to do finer-grained analysis obtaining better results. As a matter of fact, the multiple layers in deep neural networks allow models to become more efficient at learning complex features and performing more intensive computational tasks, i.e., execute many complex operations simultaneously. This is due to the ability of deep learning models to eventually learn from own errors, considering the deep learning model ability yo verify the accuracy of its predictions/outputs and make the necessary adjustments.

The paper continues in the following way: next section presents the proposed method, experiments are presented in “The evaluation” section; in “Discussion” section a state-of-the-art discussion about the adoption of artificial intelligence for pulmonary disease detection with a specific focus on *covid-19* disease is provided, and, finally, in the last section, conclusion and future research plan are drawn.

## Materials and method

The typical image classification problem is one of the most popular tasks in deep learning. It basically consists of classifying images that contain items or generic shapes (as, for instance, typewritten letters) with the highest accuracy possible. The deep learning model completes the task by leveraging the information of a dataset of input samples. In the training phase, the deep learning models extract and memorise features and patterns peculiar to a specific output class, thus learning how to distinguish between the different input samples.

One of the most widely used deep learning models for image classification is the Convolutional Neural Network (CNN), which exploit mathematical convolutional operators on the input image to extract features. The input images pass through several layers of convolution, to combines the pixels with the neighbouring ones, and subsampling, to reduce the size of the two-dimensional matrix while preserving the most relevant information. Finally, the last part of the CNN is usually composed of dense layers, which are formed by a variable number of *perceptrons*); this last part of the model perform the classification, and it is trainable with the standard backpropagation algorithm. We refer to the literature for further information on the CNNs^30,31^.

Many complex CNN variants were proposed in the literature; mainly, they differ in the size of the architecture and the number of convolutional layers. In this paper, we experiment with a CNN designed by the authors.

**Figure 1:**
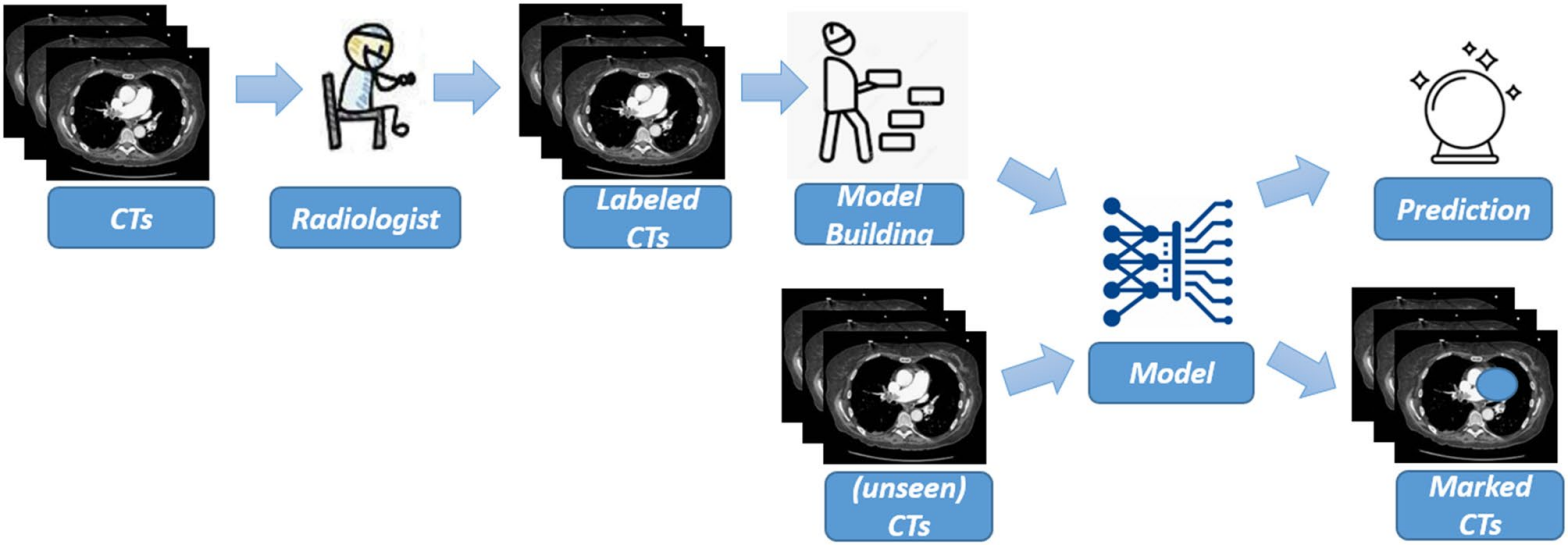
The workflow of the proposed method.

Figure 1 shows the main architecture of the approach we propose for *covid-19* detection starting from CT analysis.

We obtained a data-set of CTs, obtained from Italian hospitals (Italy was one of the first European countries where *covid-19* has widely spread^15^), belonging to different patients afflicted by different pulmonary diseases (including the *covid-19* coronavirus). Moreover also healthy patients are considered as a matter of fact the idea behind the proposed method is to infer a model, directly from the patients data, aimed to automatically identify COVID-19 patients. For this reason we need patients afflicted by *covid-19*, but also of patients afflicted from other pulmonary diseases and healthy ones, to make able to deep learning network to infer the distinctive characteristics of *covid-19* infection.

The CTs collected are scrupulously verified by expert radiologists with the aim to add a label to each patient (i.e., *COVID-19* is for a patient afflicted by the new coronavirus, *other* for patients diagnosed with other pulmonary disease and *healthy* for patient not afflicted by pulmonary diseases).

Once obtained the labelled patient CTs we input a deep learning network designed by authors, in detail we apply transfer learning.

We resort to transfer learning with fine-tuning for model training. In a nutshell we initialize the VGGNet network by exploiting weights pre-trained on the ImageNet dataset, cutting out the head related to the fully connected layer. An additional fully-connected layer head is added. It consists of several layers: AveragePooling2D, Flatten, Dense, Dropout and , finally, a Dense layer exploiting the “softmax” activation devoted to label prediction^32^. We add it above VGG16. We then freeze the VGG16’s convolutional weights in such a way that only the fully connected level head is trained and this last step completes our model configuration.

The first layer is represented by convolutional of a size fixed to 224 × 224 RGB image, for this reason CT slices are resized to this dimension. The lung CT slice is passed through a series of convolutional layers: the convolution is set to 1 pixel; the spatial padding of convolutional layer input is such that the spatial resolution is preserved after convolution. Spatial pooling is performed by exploiting 4 max-pooling layers, which follow some of the convolutional layers. Max-pooling is performed considering a two pixels high and two pixels wide window.

The first layer is represented by a convolutional layer of a fixed dimension on an RGB image of 224 × 224, this is the reason why the CT slices are resized at this height and width. The lung CT slice is passed through a series of convolutional layers: the convolution is set to 1 pixel; the spatial padding of the convolutional level input is such that the spatial resolution is preserved after the convolutional operation. Spatial pooling is performed using 4 max-pooling layers, which follow some of the convolutional layers. Max-pooling considers a window of two pixels high and two pixels wide.

All the considered hidden layers consider a rectification non-linearity. More details on the VGG-16 model architecture can be found in reference^33^.

**Figure 2:**
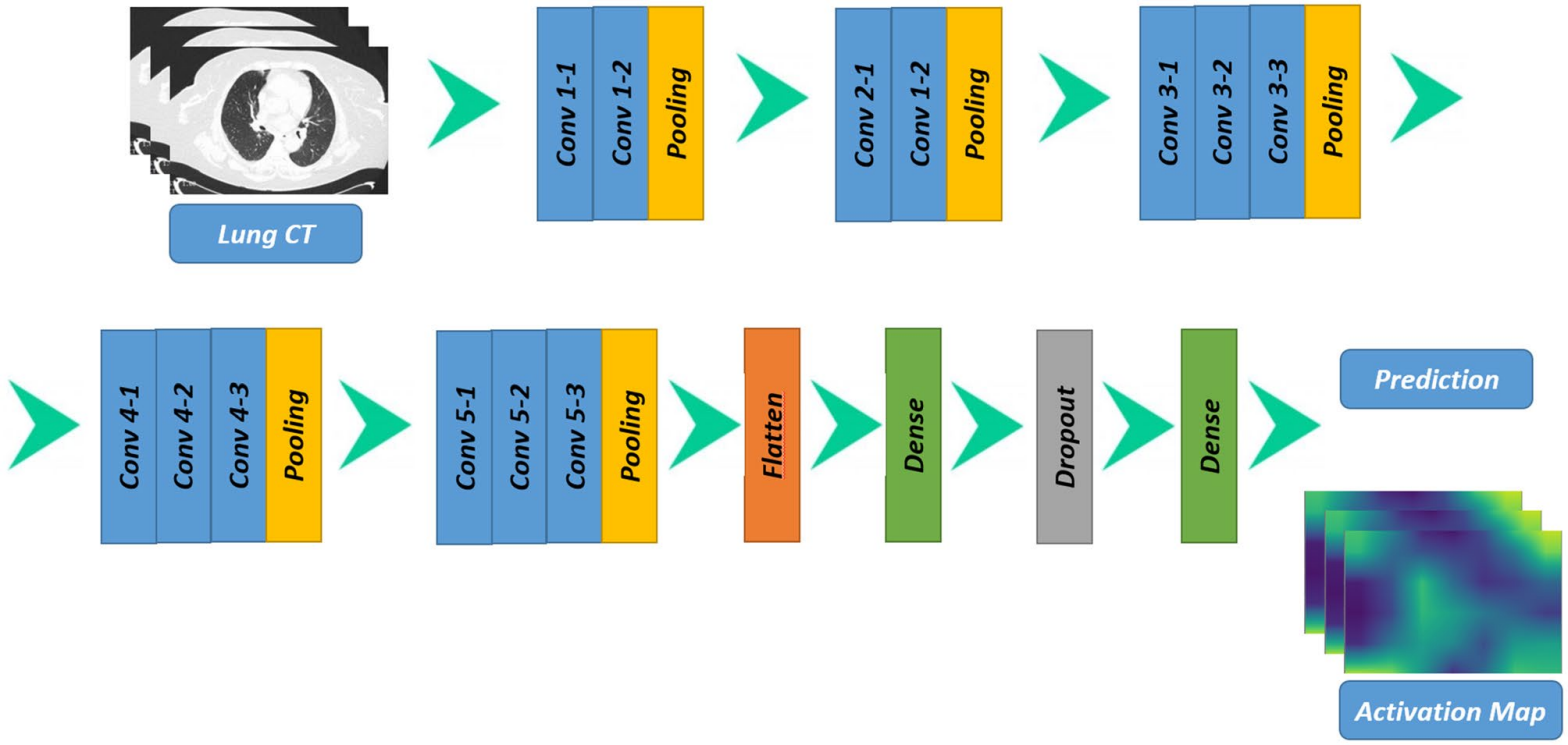
The proposed deep neural network for COVID-19 explainable detection.

We add to the VGG-16 architecture a set of layers (as shown by the deep learning model we depicted in Fig. 2): *AveragePooling2D*, *Flatten*, *Dense*, *Dropout* and another *Dense* layer.

In detail:

- *AveragePooling2D*: this layer is aimed to perform medium pooling operations. This level involves averaging for each patch of the feature map being analyzed. This means that each two pixels high and two pixels wide square of the feature map is sampled at the mean value;
- *Flatten*: the purpose of this layer is to flatten the input. It is considered a level of utility: it transforms an input, for example a row × column matrix, into a simple vector output in the form of rows * columns. Flattening transforms a two-dimensional array of features into a vector that can be inserted into a fully connected neural network classifier;
- *Dense*: is the regular deeply connected neural network layer. It is aimed to data transformation. It is most common and frequently used layer. It this case this case layer reduces the vector of height 512 to a vector of 64 elements;
- *Dropout*: this layer basically works in the following way, namely by randomly selecting neurons not considered in the training. The purpose of this level is to improve generalization, in fact we are forcing the network to train the same high-level concept by exploiting different neurons. We chose to ignore the 50% of neurons. We are aware that typically exploiting this level may result in worse performance, but we want to generate a model that is less sensitive to changes in the data;
- *Dense*: the last dense layer is aimed to reduce the vector of height 64 to a vector of 2 elements (i.e., the classes to predict).

Further details on the VGG-16 model architecture can be found in reference^33^.

To furnish explainability^4^, we resort to a visualisation considering activation maps, typically exploited for deep learning network debug. For this purpose, we resort to the Gradient-weighted Class Activation Mapping (Grad-CAM) algorithm^34^.

The Grad-CAM is a technique to extract the gradients of the DL models convolutional layers and use them to provide graphical information on the inference step. Briefly, the gradients capture high-level visual patterns and can describe which areas of the input image have influenced the most the model output decision. Also, the convolutional layer preserves spatial information, thus, the Grad-CAM uses this data to provide a heatmap of the input image. This heatmap highlights the input image area which was used by the DL model to classify a specific input; it provides a visual “explanation” to a certain decision. The Grad-CAM adopted in this work is an implementation of the one introduced by this paper^34^.

Simply put, Grad-CAM uses the gradients of any target concept, flowing into the final convolutional layer to produce a coarse location map that highlights the important regions in the image to predict the concept.

By exploiting the Grad-CAM, it is possible to validate in a visual and immediate way where the network is looking at where a chest X-ray is evaluated: in this way it is possible to verify that it is actually looking at the right patterns in the image and activating around those patterns.

Grad-CAM works by observing the last network convolutional layer and then examining the gradient information flowing into that layer. The output of the Grad-CAM is a heatmap visualization for a given predicted label. We consider the generated heatmap to visually verify where the convolutional neural network is looking in the image, as shown in the experimental analysis section.

## The evaluation

In the follow we present the results we obtained by the experimental analysis.

As stated into the previous section, we gathered a dataset to validate the effectiveness of the proposed method. We obtain CTs and the radiologist diagnosis for the following 45 patients:

- 20 patients afflicted by *covid-19* disease;
- 9 patients afflicted by *other* pulmonary diseases;
- 16 patients without pulmonary diseases, labelled as *healthy*.

The proposed method marked as *covid-19* 19 patients on 20 afflicted by COVID-19. With regard to the 9 patients afflicted by other pulmonary diseases only 1 was wrongly labelled as *covid-19*, while the remaining ones were rightly labelled as *non covid-19*. All the 16 *healthy* were rightly labelled as *non covid-19* by the proposed method. For each patient, in average, we have 400 images, for an average total number equal to 18,000 images. The medical images were obtained from the Department of Precision Medicine, belonging to the University of Campania, Caserta, Italy.

The 400 central images of the CT examination were considered for each patient, thus excluding the totally black ones that did not present any organ.

We train the designed deep learning network by considering the 80% of the dataset, while the remaining 20% is exploited for the model testing. Balanced instance were considered for the three classes involved in the experiment (i.e., covid-19, other and healthy), with a cross-validation with k = 5 in order to evaluate all the patients.

In k-fold cross validation, the dataset is divided into a series of equal portions of data (k-fields) and a set of images belonging to the dataset is exploited for the training, while the remaining is exploited for the testing. The cross-validation is a technique for training a model on subsets of the available input data and evaluating them against a complementary subset of the data. We resort to cross-validation to avoid overfitting, which is the failure to generalize a model. In practice, the k-fold cross-validation method divides the input data into k subsets of data (also known as folds). We train a model with (k-1) subsets of data, then we evaluate the model on the subset that was not used for training i.e., the remaining one. This process is repeated k times, each time with a different subset reserved for evaluation (and clearly excluded from training). In the experimental analysis we selected k = 5: in this way the dataset was split into 5 equal parts, where each part contains the 20% of the images belonging to the dataset. Starting from this, 5 different model training were carried out, where for each training the 80% of the dataset (therefore 4 parts out of 5) was considered for training and the remaining 20% for testing. In subsequent training, the remaining 20% was used for testing. The final performance values are therefore the average of the performance values obtained in the 5 classifications, where a 20% (different for each classification) of the dataset was used in each classification.

Table 1 shows the performance obtained by the proposed method in terms of sensitivity, specificity, f-measure and accuracy.

**Table 1:** Experimental analysis evaluation.

Model	Sensitivity	Specificity	F-measure	Accuracy
Our method	0.95	0.95	0.95	0.95

As shown from the experimental analysis evaluation in Table 1, a sensitivity and a specificity equal to 0.95 is reached with regard to the discrimination between patients afflicted by *covid-19* disease, patient afflicted by other pulmonary diseases and healthy ones.

**Figure 3:**
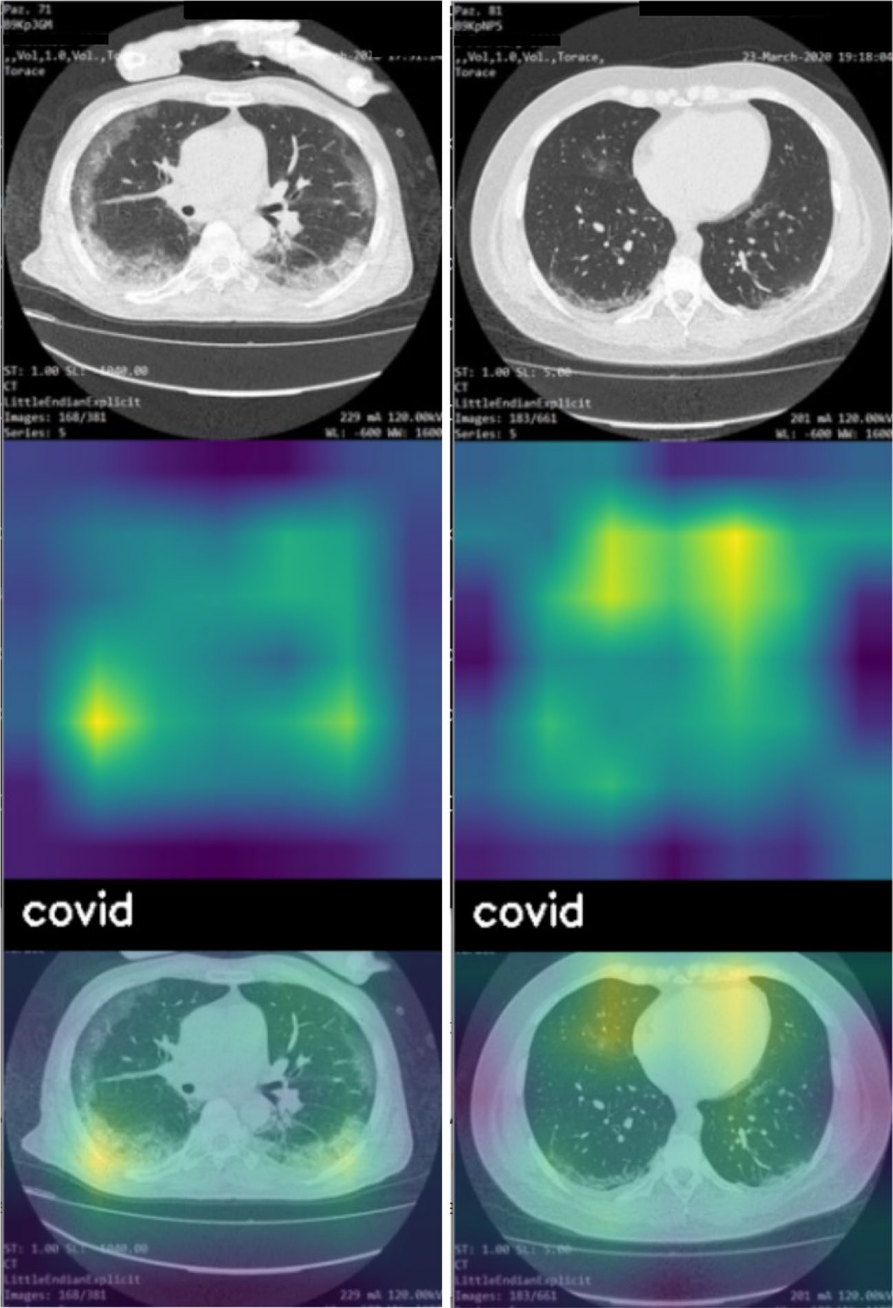
Two slices belonging to two different *covid-19* patients with the related activation maps: in yellow the areas symptomatic of the *covid-19* infection.

Figure 3 provides a couple of example of explainability automatically inferred by the proposed approach. In fact, we show two slices (belonging to two different *covid-19* patients as shown from the labels applied by the deep learning model), the activation maps and the overlay between the slices and the activation maps with the aim to show the area of interest (highlighted in yellow and green, while the blue area are not considered of interest from the model to perform the detection).

From the activation map related to the first slice (in the left part of Fig. 3) we note that the low areas of the CT are symptomatic of the *covid-19* disease infection, while in the second slice (in the right part of Fig. 3) it seems that the upper areas are more symptomatic of the *covid-19* disease infection. The overlay of the activation maps with the slices provide explainability about the relevant areas symptomatic of the *covid-19* disease. Moreover, they can provide interesting insights for radiologists for the localisation of the areas interested by the *covid-19* infection. The outcomes of the activation maps were confirmed also by expert radiologists.

The analysis requires for a new CT series approximately 8.9 s to make the prediction and the visualise the activation maps. The machine used to run the experiments and to take measurements was an Intel Core i7 8th gen, equipped with 2GPU and 16Gb of RAM.

## Discussion

Current literature presents several research papers focused to *covid-19* detection. Below we discuss these papers.

Authors in^35^ exploit deep learning obtaining an accuracy equal to 98.85%. The main difference with respect to the proposed method is that authors in^35^ do not take into account healthy patients. Moreover they do not provide explainability about their network predictions.

Chen et al.^35^ considering a deep network achieving an accuracy of 98.85%. The main difference from the proposed method is that authors in^35^ do not consider patients without pulmonary pathologies. They also do not provide explainability about the deep learning predictions.

Wang et al.^36^ design a deep learning model for detecting *covid-19*. The authors do not consider patients without pulmonary pathologies and do not provide some sort of explanation on the results of their network. Additionally, the method in^35^ requires you to manually tag the *covid-19* region of interest. They obtained accuracy of 73.1% by evaluating a dataset obtained from two hospitals.

Xu and colleagues^37^ propose a deep learning approach obtaining a 86.7% accuracy. Two three-dimensional convolutional neural networks are exploited: the first is the ResNet23 network, while the second network represents a variant of the first, in which the authors have added several layers.

Below, we discuss research works^38,39^ on the application of deep learning techniques to proteins with the aim of promoting the study for new vaccines.

Zhang et al.^39^ leverages deep learning techniques by exploiting *covid-19* RNA sequences to predict which current antivirals may help *covid-19* patients.

Beck et al.^38^ experiment the adoption of deep models in combination with a molecule repository. The outcome of this research is that 2019-nCoV 3C proteinase is expected to bind with atazanavir, which is an antiviral drug used to treat Human Immunodeficiency Virus.

Apostolopoulos and colleagues^40^ exploit transfer learning reaching a 0.98 detection rate in the discrimination between *covid-19* and healthy patient.

Zheng and colleagues in reference^41^ adopt a model for detecting *covid-19* from CT images reaching a detection rate equal of 0.9, while researchers in^37^ obtain a 0.86 detection rate in *covid-19* identification exploiting the ResNet network by analyzing CT images. Most of these articles , with respect to the proposed work, evaluate a small amount of data for model training and they do not provide explainability of the results.

Narin et al. in^42^ propose the usage of 3 networks evaluating 50 *covid-19* chest X-ray images and 50 heartily images gathered from a Kaggle competition (https://www.kaggle.com/paultimothymooney/chest-xray-pneumonia). It should be noted that in the reference^42^ researchers consider non-COVID images belonging to children, while those *covid-19* are related to adult patients.

Researchers in^43^ achieved an accuracy equal to 0.86 analysing CT images with a deep model built on the ResNet50 model. Wang et al.^36^ achieved an accuracy of 0.82 exploiting the modified Inception (M-Inception) deep model by analysing CT images.

Song et al.^43^ reached a 0.86 accuracy by analyzing CT images with a deep learning network built on the ResNet50 model, while researchers in^36^ reached a 0.82detection rate by exploiting the Inception network with CT medical images.

Ozturk and colleagues^44^ analyse 1750 x-ray adopting the DarkCovidNet network: in detail they consider250 *covid-19* positive patient, 500 related to generic lung diseases and 1000 obtained from healthy patients obtaining a detection rate equal to 0.98.

Ardakani et al.^45^ propose a deep learning based approach for distinguishing from *covid-19* from non-*covid-19*. Ten different well-known convolutional neural networks were used. Using the Xception model they obtained an accuracy equal to 0.99.

Researchers in^45^ design a method aimed to discriminate between *covid-19* and non-*covid-19* patients. Ten deep learning models are considered for this purpose and a detection rate equal to 0.99 is obtained with the Xception network.

Authors in^18^ leveraged the COVNet model to identify the *covid-19* by learning a RestNet50 models, by reaching a detection rate of 0.96. Also this method, differently from the one we proposed, does not provide prediction explainability.

Transfer learning is exploited also in^4^ for covid detection. Differently from the proposed method, in reference^4^ the analysis is performed on x-ray images.

Authors in^46^ propose a method aimed to detect finger skin. They exploit three kinds of images: 60 h after injury, 160 h after injury, 450 h after injury. Authors state that the advantage of the presented method is the automatic detection of the finger skin using a smartphone and they method can be helpful to diagnose pathologies of human skin.

Piekarski and colleagues^47^ exploit a CNN for fault detection in time series data. In a nutshell, they propose a method aimed to detect the abnormal status of sensors in certain time steps. They consider transfer learning by examining pre-trained VGG-16, VGG-19, InceptionV3 and Xception CNN models with an adjusted densely-connected classifiers.

In Table 2 we compare the current state-of-the-art in COVID detection by means of deep learning in terms of acquisition (X-ray or CT), number of images analysis (Images column) and the obtained accuracy.

**Table 2:** State-of-the-art comparison.

Method	Acquisition	Images	Accuracy
Apostolopoulos et al.^40^	X-ray	1428	0.93
Wang and Wong^48^	X-ray	13,645	0.92
Sethy et al.^49^	X-ray	50	0.95
Hemdan et al.^50^	X-ray	50	0.90
Narin et al.^42^	X-ray	100	0.98
Song et al.^43^	CT	1485	0.86
Wang et al.^36^	CT	453	0.82
Zheng et al.^41^	CT	542	0.90
Xu et al.^37^	X-ray	618	0.86
Ozturk et al.^44^	X-ray	1750	0.92
Ardakani et al.^45^	CT	1020	0.99
Li et al.^18^	CT	4356	0.96
Butt et al.^51^	CT	1710	0.86
Brunese et al.^4^	X-ray	6523	0.97
Our method	CT	18,000	0.95

Moreover in Table 3 we compare four of the most exploited deep learning model used in covid-19 detection^52^ with the dataset we gathered. The aim of this experiment is to perform a direct comparison between the deep learning models currently employed by the state-of-the-art and the proposed model.

**Table 3:** Experimental comparison.

Model	Sensitivity	Specificity	F-measure	Accuracy
ResNet50	0.89	0.91	0.82	0.92
VGG19	0.90	0.92	0.83	0.93
AlexNet	0.72	0.69	0.69	0.71
InceptionV3	0.84	0.82	0.81	0.84
Our method	0.95	0.95	0.95	0.95

As shown from the experimental results obtained with the proposed model in Table 1, the state-of-the-art models obtain an accuracy ranging from 0.71 to 0.93, while the proposed method is able to reach an accuracy equal to 0.95, thus confirming the effectiveness of the proposed method.

**Figure 4:**
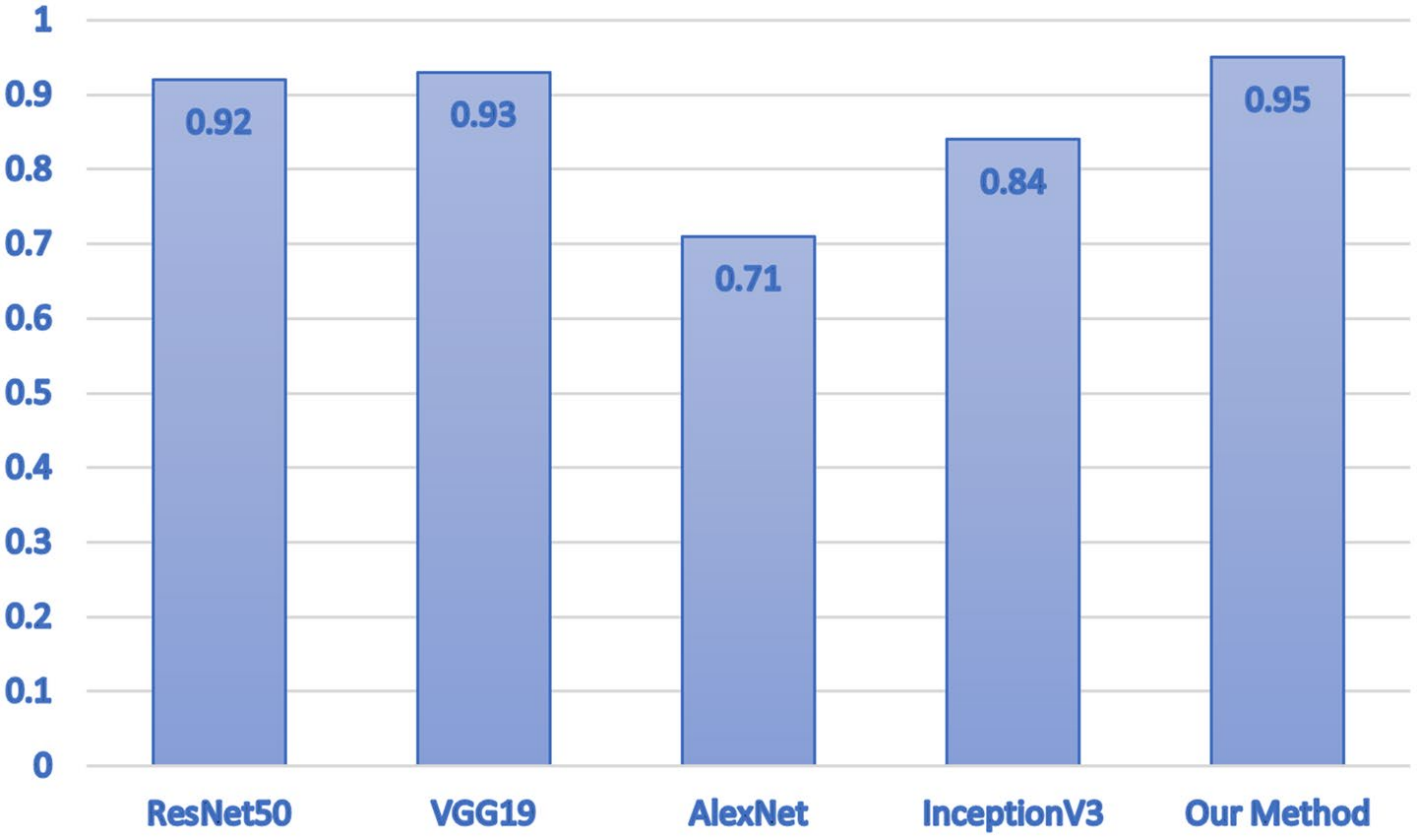
The comparison in terms of accuracy obtained by the state-of-the-art models and the proposed deep learning model.

In Fig. 4 we report a chart aimed to present a direct visual comparison between the state-of-the-art models and the proposed deep learning model in terms of accuracy.

From Fig. 4 it emerges that the model obtaining the worst performances is the AlexNet one, the InceptionV3 model obtains slightly better performances, the ResNet50 and VGG19 models obtain a good interesting and, finally, the proposed model with an accuracy of 0.95 overcomes the performances of the remaining analysed models.

## Conclusion and future work

We design an approach focused on the detection of *covid-19* by analysing CT medical images. In particular, we consider a transfer learning model developed by authors, aimed to label a CT as *covid-19*, for patients affected with the *covid-19*; *other*, for patients affected with a pulmonary disease different from the *covid-19* one; and *healthy*, for patients with no pulmonary disease. As additional contribution, the proposed approach is aimed to highlight on the patient CT the areas symptomatic of the infection provided by *covid-19*: this represents an important characteristic because this representation can provide interesting and useful information to pathologist and radiologist. We think that the proposed method can be considered for a rapid screening and for an immediate diagnosis and visualisation of the lung areas affected by the *covid-19* infection.

As a matter of fact, the GRAD-CAM was applied to other contexts, for instance cybersecurity^53^ for this reason, we are confident that the proposed method can be exploited for explainable classification tasks in other contexts.

Furthermore, we will explore if model checking can be considered to increase the novel coronavirus detection accuracy obtained by the proposed method: as a matter of fact, model checking already demonstrated their effectiveness in medical context as, for instance, the detection of prostate cancer Gleason score from computed tomography images^54^.

## Data Availability

The dataset used and analysed during the current study is available from the corresponding author on reasonable request.

## References

[1] Struyf T (2022). Signs and symptoms to determine if a patient presenting in primary care or hospital outpatient settings has COVID-19. Cochrane Database Syst. Rev..

[2] Brunese L, Martinelli F, Mercaldo F, Santone A (2020). Machine learning for coronavirus COVID-19 detection from chest x-rays. Procedia Comput. Sci..

[3] Jeyanathan M, Afkhami S, Smaill F, Miller MS, Lichty BD, Xing Z (2020). Immunological considerations for COVID-19 vaccine strategies. Nat. Rev. Immunol..

[4] Brunese L, Mercaldo F, Reginelli A, Santone A (2020). Explainable deep learning for pulmonary disease and coronavirus COVID-19 detection from x-rays. Comput. Methods Programs Biomed..

[5] Le TT, Andreadakis Z, Kumar A, Roman RG, Tollefsen S, Saville M, Mayhew S (2020). The COVID-19 vaccine development landscape. Nat. Rev. Drug Discov..

[6] Li Q (2020). Early transmission dynamics in Wuhan, China, of novel coronavirus-infected pneumonia. New Engl. J. Med..

[7] Gu J, Han B, Wang J (2020). COVID-19: Gastrointestinal manifestations and potential fecal-oral transmission. Gastroenterology.

[8] Roques L, Klein EK, Papaix J, Sar A, Soubeyrand S (2020). Using early data to estimate the actual infection fatality ratio from COVID-19 in France. Biology.

[9] Covid, T.C., Team, R (2020). Severe outcomes among patients with coronavirus disease 2019 (COVID-19)-United States. MMWR Morb. Mortal. Wkly. Rep..

[10] Wang Y, Wang Y, Chen Y, Qin Q (2020). Unique epidemiological and clinical features of the emerging 2019 novel coronavirus pneumonia (COVID-19) implicate special control measures. J. Med. Virol..

[11] Holmes KV (2003). SARS-associated coronavirus. N. Engl. J. Med..

[12] van der Hoek L, Pyrc K, Jebbink MF, Vermeulen-Oost W, Berkhout RJ, Wolthers KC, Wertheim-van Dillen PM, Kaandorp J, Spaargaren J, Berkhout B (2004). Identification of a new human coronavirus. Nat. Med..

[13] Abroug F, Slim A, Ouanes-Besbes L, Kacem M-AH, Dachraoui F, Ouanes I, Lu X, Tao Y, Paden C, Caidi H (2014). Family cluster of middle east respiratory syndrome coronavirus infections, Tunisia, 2013. Emerg. Infect. Dis..

[14] Jung S-M, Akhmetzhanov AR, Hayashi K, Linton NM, Yang Y, Yuan B, Kobayashi T, Kinoshita R, Nishiura H (2020). Real-time estimation of the risk of death from novel coronavirus (COVID-19) infection: Inference using exported cases. J. Clin. Med..

[15] Livingston E, Bucher K (2020). Coronavirus disease 2019 (COVID-19) in Italy. Jama.

[16] Wang C, Horby PW, Hayden FG, Gao GF (2020). A novel coronavirus outbreak of global health concern. Lancet.

[17] Huang C, Wang Y, Li X, Ren L, Zhao J, Hu Y, Zhang L, Fan G, Xu J, Gu X (2020). Clinical features of patients infected with 2019 novel coronavirus in Wuhan, China. Lancet.

[18] Li L (2020). Artificial intelligence distinguishes COVID-19 from community acquired pneumonia on chest CT. Radiology.

[19] Long C, Xu H, Shen Q, Zhang X, Fan B, Wang C, Zeng B, Li Z, Li X, Li H (2020). Diagnosis of the coronavirus disease (COVID-19): RRT-PCR or CT?. Eur. J. Radiol..

[20] Sellers SA, Dover KL, Bailey AG, Cheves A, Eason AB, Popowitch EB, Miller MB, Wohl DA, Dittmer DP, Fischer WA (2020). Burden of respiratory viral infection in persons with human immunodeficiency virus. Influenza Other Respir. Viruses.

[21] Ai T (2019). Correlation of chest CT and RT-PCR testing in coronavirus disease 2019 (COVID-19) in China: A report of 1014 cases. Radiology.

[22] Fang Y, Zhang H, Xie J, Lin M, Ying L, Pang P, Ji W (2020). Sensitivity of chest CT for COVID-19: Comparison to RT-PCR. Radiology.

[23] Alakwaa W, Nassef M, Badr A (2017). Lung cancer detection and classification with 3D convolutional neural network (3D-CNN). Lung Cancer.

[24] Bhatia S, Sinha Y, Goel L (2019). Lung cancer detection: A deep learning approach. Soft Computing for Problem Solving.

[25] Brunese, L., Mercaldo, F., Reginelli, A., & Santone, A. Neural networks for lung cancer detection through radiomic features. In *2019 International Joint Conference on Neural Networks (IJCNN)*, 1–10 (IEEE, 2019).

[26] Bulten W, Pinckaers H, van Boven H, Vink R, de Bel T, van Ginneken B, van der Laak J, Hulsbergen-van de Kaa C, Litjens G (2020). Automated deep-learning system for Gleason grading of prostate cancer using biopsies: A diagnostic study. Lancet Oncol..

[27] Puderbach M, Eichinger M, Haeselbarth J, Ley S, Kopp-Schneider A, Tuengerthal S, Schmaehl A, Fink C, Plathow C, Wiebel M (2007). Assessment of morphological MRI for pulmonary changes in cystic fibrosis (CF) patients: Comparison to thin-section CT and chest X-ray. Investig. Radiol..

[28] Rohde M, Nielsen AL, Johansen J, Sørensen JA, Nguyen N, Diaz A, Nielsen MK, Asmussen JT, Christiansen JM, Gerke O (2017). Head-to-head comparison of chest x-ray/head and neck MRI, chest CT/head and neck MRI, and 18F-FDG PET/CT for detection of distant metastases and synchronous cancer in oral, pharyngeal, and laryngeal cancer. J. Nucl. Med..

[29] Schaefer O, Langer M (2007). Detection of recurrent rectal cancer with CT, MRI and PET/CT. Eur. Radiol..

[30] Khan S, Rahmani H, Shah SAA, Bennamoun M (2018). A guide to convolutional neural networks for computer vision. Synth. Lect. Comput. Vis..

[31] LeCun Y, Bottou L, Bengio Y, Haffner P (1998). Gradient-based learning applied to document recognition. Proc. IEEE.

[32] de Lima Hedayioglu, F., Coimbra, M. T., & da Silva Mattos, S. A survey of audio processing algorithms for digital stethoscopes. In *HEALTHINF* 425–429, (2009).

[33] Simonyan, K. & Zisserman, A. Very deep convolutional networks for large-scale image recognition. arXiv preprint arXiv:1409.1556, (2014).

[34] Selvaraju, R. R. *et al.* Grad-cam: Visual explanations from deep networks via gradient-based localization. In *Proceedings of the IEEE International Conference on Computer Vision* 618–626, (2017).

[35] Chen J, Wu L, Zhang J (2020). Deep learning-based model for detecting 2019 novel coronavirus pneumonia on high-resolution computed tomography. Sci Rep.

[36] Wang S, Kang B, Ma J, Zeng X, Xiao M, Guo J, Cai M, Yang J, Li Y, Meng X (2021). A deep learning algorithm using CT images to screen for corona virus disease (COVID-19). Eur. Radiol..

[37] Xu X, Jiang X, Ma C, Du P, Li X, Lv S, Yu L, Ni Q, Chen Y, Su J (2020). A deep learning system to screen novel coronavirus disease 2019 pneumonia. Engineering.

[38] Beck BR, Shin B, Choi Y, Park S, Kang K (2020). Predicting commercially available antiviral drugs that may act on the novel coronavirus (2019-nCoV), Wuhan, China through a drug-target interaction deep learning model. bioRxiv.

[39] Zhang, H. *et al.* Deep learning based drug screening for novel coronavirus 2019-nCoV. (2020). 10.1007/s12539-020-00376-6 PMC7266118 32488835

[40] Apostolopoulos ID, Mpesiana TA (2020). Covid-19: Automatic detection from x-ray images utilizing transfer learning with convolutional neural networks. Phys. Eng. Sci. Med..

[41] Wang, X. *et al.* A weakly-supervised framework for COVID-19 classification and lesion localization from chest CT. In *IEEE Transactions on Medical Imaging*, **39**(8), 2615–2625 10.1109/TMI.2020.2995965 (2020). 10.1109/TMI.2020.2995965 33156775

[42] Narin, A., Kaya, C., & Pamuk, Z. Automatic detection of coronavirus disease (COVID-19) using x-ray images and deep convolutional neural networks. arXiv preprint arXiv:2003.10849, (2020). 10.1007/s10044-021-00984-y PMC8106971 33994847

[43] Song Y, Zheng S, Li L, Zhang X, Zhang X, Huang Z, Chen J, Wang R, Zhao H, Chong Y (2021). Deep learning enables accurate diagnosis of novel coronavirus (COVID-19) with CT images. IEEE/ACM Trans. Comput. Biol. Bioinform..

[44] Ozturk T (2020). Automated detection of COVID-19 cases using deep neural networks with x-ray images. Comput. Biol. Med..

[45] Ardakani AA, Kanafi AR, Acharya UR, Khadem N, Mohammadi A (2020). Application of deep learning technique to manage COVID-19 in routine clinical practice using CT images: Results of 10 convolutional neural networks. Comput. Biol. Med..

[46] Glowacz A, Glowacz Z (2016). Recognition of images of finger skin with application of histogram, image filtration and K-NN classifier. Biocybern. Biomed. Eng..

[47] Piekarski M, Jaworek-Korjakowska J, Wawrzyniak AI, Gorgon M (2020). Convolutional neural network architecture for beam instabilities identification in synchrotron radiation systems as an anomaly detection problem. Measurement.

[48] Wang L, Lin ZQ, Wong A (2020). Covid-net: A tailored deep convolutional neural network design for detection of covid-19 cases from chest x-ray images. Sci. Rep..

[49] Sethy, P. K. & Behera, S. K. Detection of coronavirus disease (COVID-19) based on deep features. (2020).

[50] Hemdan, E. E.-D., Shouman, M. A., & Karar, M. E. Covidx-net: A framework of deep learning classifiers to diagnose COVID-19 in x-ray images. arXiv preprint arXiv:2003.11055, (2020).

[51] Butt C, Gill J, Chun D, Babu BA (2019). Deep learning system to screen coronavirus disease, pneumonia. Appl. Intell..

[52] Peláez E, Serrano R, Murillo G, Cárdenas W (2021). A comparison of deep learning models for detecting covid-19 in chest x-ray images. Ifac-papersonline.

[53] Iadarola G, Martinelli F, Mercaldo F, Santone A (2021). Towards an interpretable deep learning model for mobile malware detection and family identification. Comput. Security.

[54] Brunese L, Mercaldo F, Reginelli A, Santone A (2019). Prostate Gleason score detection and cancer treatment through real-time formal verification. IEEE Access.

